# Non-Peptidic Small Molecule Components from Cone Snail Venoms

**DOI:** 10.3389/fphar.2021.655981

**Published:** 2021-05-13

**Authors:** Zhenjian Lin, Joshua P. Torres, Maren Watkins, Noemi Paguigan, Changshan Niu, Julita S. Imperial, Jortan Tun, Helena Safavi-Hemami, Rocio K. Finol-Urdaneta, Jorge L. B. Neves, Samuel Espino, Manju Karthikeyan, Baldomero M. Olivera, Eric W. Schmidt

**Affiliations:** ^1^ Departments of Medicinal Chemistry and Biochemistry, School of Biological Sciences, University of Utah, Salt Lake City, UT, United States; ^2^ Faculty of Health and Medical Sciences, Department of Biomedical Sciences, University of Copenhagen, Copenhagen, Denmark; ^3^ Illawarra Health and Medical Research Institute, University of Wollongong, Wollongong, NSW, Australia; ^4^ Interdisciplinary Centre of Marine and Environmental Research, CIIMAR/ CIMAR, Faculty of Sciences, University of Porto, Porto, Portugal

**Keywords:** secondary metabolites, conus, gastropod, prey capture, conopeptides, natural products, venom, nicotinic acetylcholine receptor

## Abstract

Venomous molluscs (Superfamily Conoidea) comprise a substantial fraction of tropical marine biodiversity (>15,000 species). Prior characterization of cone snail venoms established that bioactive venom components used to capture prey, defend against predators and for competitive interactions were relatively small, structured peptides (10–35 amino acids), most with multiple disulfide crosslinks. These venom components (“conotoxins, conopeptides”) have been widely studied in many laboratories, leading to pharmaceutical agents and probes. In this review, we describe how it has recently become clear that to varying degrees, cone snail venoms also contain bioactive non-peptidic small molecule components. Since the initial discovery of genuanine as the first bioactive venom small molecule with an unprecedented structure, a broad set of cone snail venoms have been examined for non-peptidic bioactive components. In particular, a basal clade of cone snails (*Stephanoconus*) that prey on polychaetes produce genuanine and many other small molecules in their venoms, suggesting that this lineage may be a rich source of non-peptidic cone snail venom natural products. In contrast to standing dogma in the field that peptide and proteins are predominantly used for prey capture in cone snails, these small molecules also contribute to prey capture and push the molecular diversity of cone snails beyond peptides. The compounds so far characterized are active on neurons and thus may potentially serve as leads for neuronal diseases. Thus, in analogy to the incredible pharmacopeia resulting from studying venom peptides, these small molecules may provide a new resource of pharmacological agents.

## Introduction

The venomous cone snails comprise a biodiverse lineage of marine gastropods (∼1,000 living species) that specialize on the spectrum of prey (fish, other gastropod molluscs, or polychaete worms) envenomated by each species. The cone snails can be grouped into distinct clades, based on molecular phylogenetic data; these divisions generally correlate with the prey specialization of each clade (Nam et al., 2009; Kraus et al., 2011). Despite the enormous range of biology evolved across the entire group, there is a general feature characteristic of the entire family (Conidae): all cone snails have complex venoms each with its own distinctive complement of typically 100–200 bioactive venom components (Olivera et al., 2014). The venoms are produced in a long venom gland, lined with secretory epithelial cells where the biosynthesis of venom components takes place. The venom is injected by extending a proboscis from the anterior gut, through a highly -specialized radular tooth that serves as a hypodermic needle (Kohn et al., 1999).

No matter what the prey type, many venom components are encoded by a few well-characterized gene superfamilies expressed in the venom glands and distributed through all of the diverse lineages of cone snails (Robinson and Norton, 2014). One such example of a well-characterized group of venom peptides are the α-conopeptides that belong to the A-gene superfamily: the bioactive post-translationally processed gene products are typically small (10–25 amino acids) peptides with two disulfide bonds (Puillandre et al., 2010). The mature peptides are encoded at the C-terminal end of a canonical precursor with a conserved signal sequence and an intervening propeptide region. The gene structure that encodes this family of venom components is conserved; a large fraction of the peptides encoded by this gene superfamily share their general targeting specificity—these inhibit nicotinic acetylcholine receptors of various types (McIntosh et al., 1999). Cone snail venom peptides of this type have now been studied for many decades and are increasingly well characterized (Azam and McIntosh, 2009).

The purpose of this article is to balance the perception that the bioactive components of cone snail venoms are all small peptides (typified by the α-conopeptides). Much less well-recognized is the emerging evidence that non-peptidic small molecules are used by certain lineages of cone snails as part of their strategy of envenomation. Since the literature is so dominated by studies of conopeptides, we review the expanding literature on small molecule venom components, and present an overview framework for one lineage within the family Conidae, the subgenus *Stephanoconus*, a well-characterized group of vermivorous cone snails. The small molecule strategy of this group is being actively investigated, and we will both review the existing literature and unpublished data, as well as provide a potential framework for future research directions.

## The *Stephanoconus* Clade: Phylogeny and Biology

**FIGURE 1:**
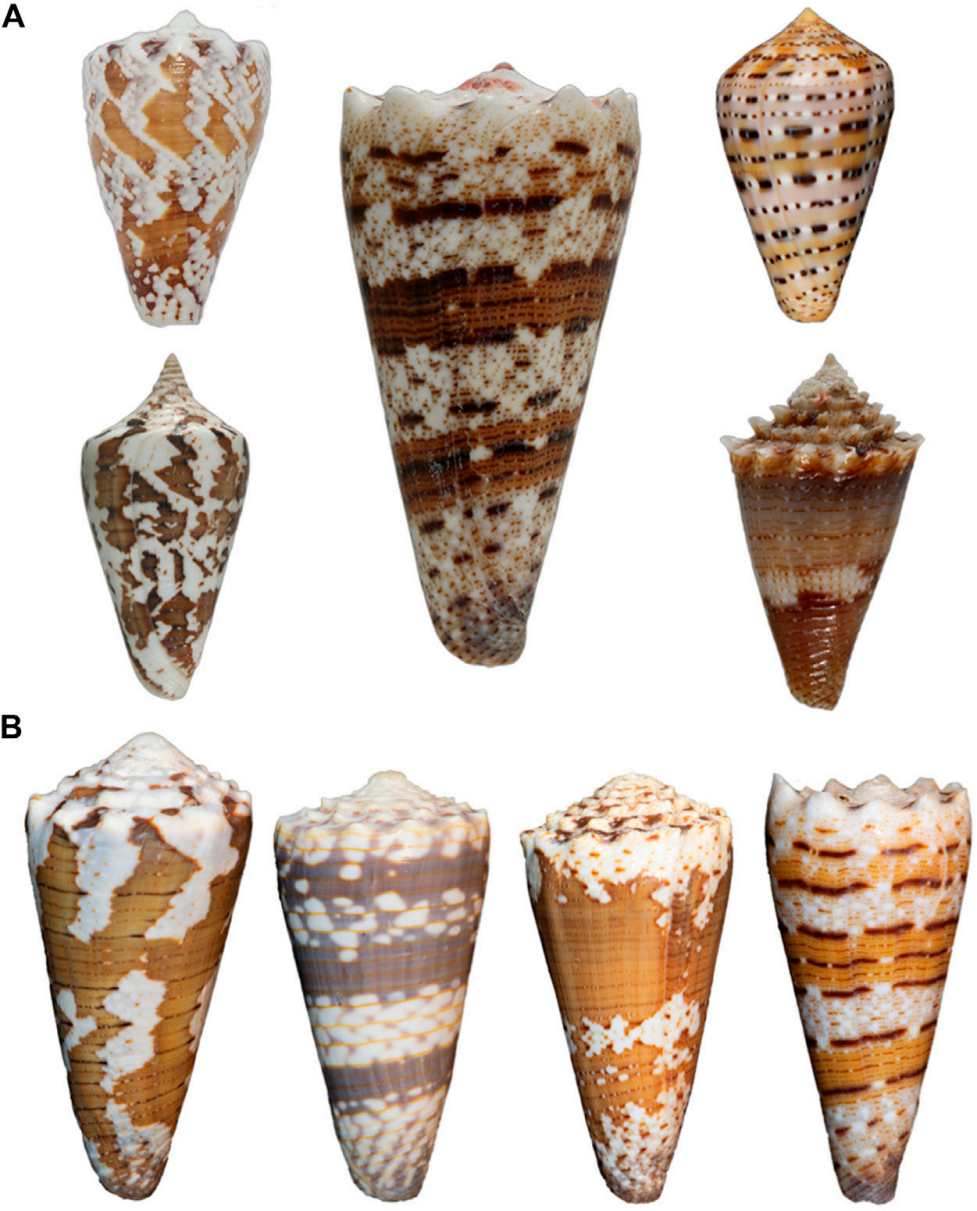
**(A)** Cone shells of *Stephanoconus* species. Left top, *Conus regius* (Florida); Left bottom, *Conus archon* (West Mexico); Center, *Conus imperialis* (Philippines); Right top, *Conus genuanus* (West Africa); Right bottom, *Conus chiangi* (Philippines, 150–250 m). **(B)** Variation in Indo-Pacific *Stephanoconus*. From left to right: *Conus imperialis fuscatus* (sometimes called *viridulus*; Zanzibar, East Africa); *Conus zonatus* (Laccadive Islands, India); *Conus imperialis* variety (Reunion Island); *Conus imperialis* variety (Balicasag Island, Philippines, gill nets, 70–120 fathoms). All four forms may represent different species from the *Conus imperialis* specimen shown in **A**, but only *Conus zonatus* is generally accepted as being different (Photography by Sam Watson and Sam Espino.)

We will describe small molecule natural products broadly across diverse cone snail groups, but the major lineage of cone snails that will be a focus of this article is the *Stephanoconus* clade. Examples of the shells of species in this clade are illustrated in Figure 1. These species are not treated in a taxonomically consistent manner in the literature — we regard *Stephanoconus* as a subgenus of *Conus*, within the family Conidae (Puillandre et al., 2014). Other workers (Tucker and Tenorio, 2009; Monnier et al., 2018) have raised *Stephanoconus* to the level of a full genus, and have variously subdivided this group into smaller units (proposing genera such as *Tenorioconus*, *Tarenteconus*, *Rhombiconus*, which we will treat as synonyms of *Stephanoconus*). Some of these proposed genera are recognized as an “alternate representation” by the World Register of Marine Organisms, while others are listed as “unaccepted” (Board, 2020). The molecular data are consistent with including all of the species in Figure 1 in a single clade, *Stephanoconus*.

**FIGURE 2:**
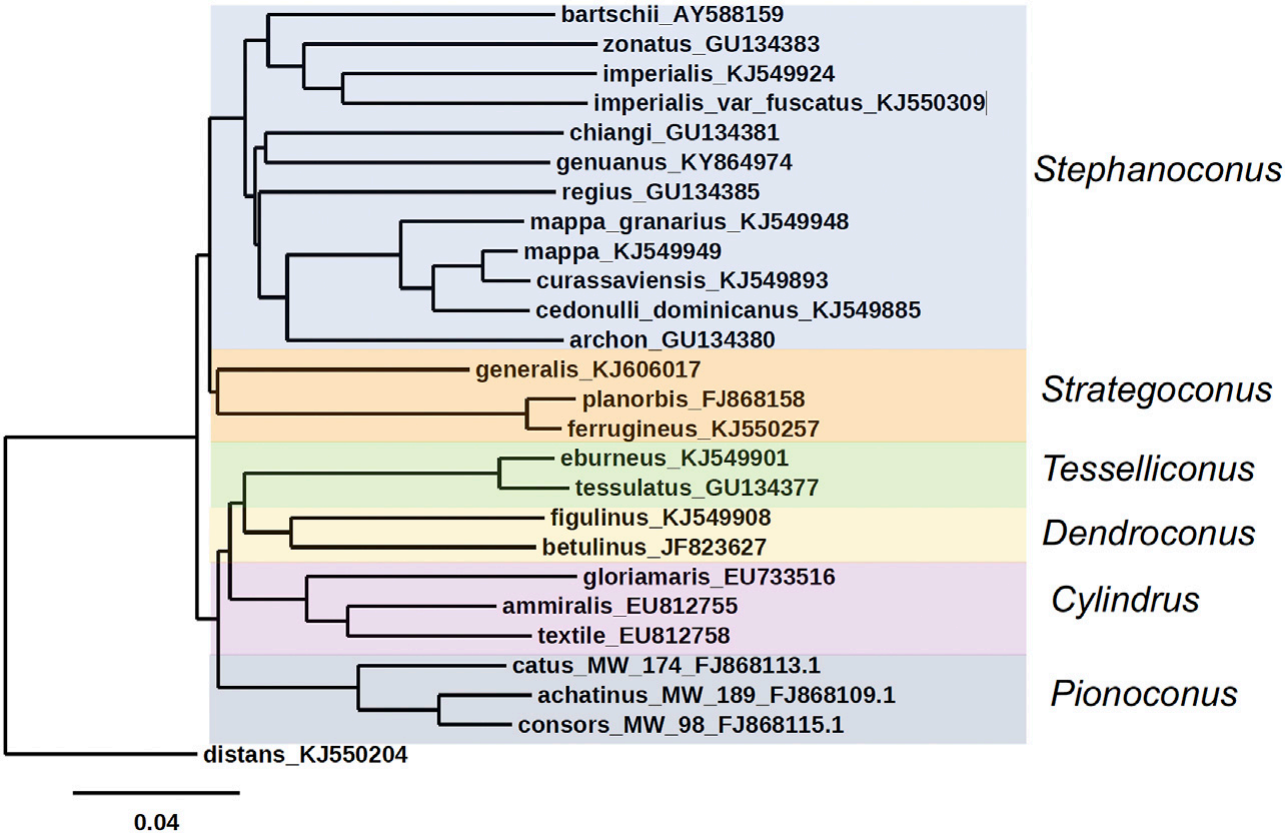
Phylogenetic tree of *Stephanoconus*, *Strategoconus*, *Dendroconu*s, *Tesselliconus* (vermivorous), *Cylindrus* (molluscivorous), and *Pionoconu*s (piscivorous) clades of *Conus* species, using mitochondrial cytochrome oxidase C (COI) marker. *Conus distans* used as outgroup. Genbank accession numbers KJ549885, KJ549949, KJ549924, KJ549948, KJ549893, KJ549901, KJ5499908, KJ550257, KJ550309, and KJ550204 (Puillandre et al., 2014); GU134380, GU134381, GU134383, and GU134385 (Watkins et al., 2010); AY588159 (Duda and Rolan, 2005); KY864974 (Abalde et al., 2019); FJ868158 (Biggs et al., 2010); KJ606017 (Aman et al., 2015); FJ868109, FJ868113, and FJ868115 (Kantor and Taylor, 2000; Puillandre et al., 2010); EU733516, EU812755, EU812758, and GU134377 (Nam et al., 2009); JF823627 (Cabang et al., 2011; Olivera et al., 2015).

An unusual feature of *Stephanoconus* is that the species included have a cosmopolitan distribution, comprising species from the Indo-Pacific, Panamic, Caribbean and Eastern Atlantic marine provinces. There is no other lineage of *Conus* with such a broad geographic distribution (Peters et al., 2013). In most phylogenetic trees, *Stephanoconus* appears as one of the basal lineages within the genus *Conus* (all of the basal lineages are specialists on different types of polychaete worms) (Duda et al., 2001). The phylogenetic tree for the whole genus *Conus* suggests that fish-hunting and mollusc-hunting groups evolved sometime in the Miocene from worm-hunting ancestors (Aman et al., 2015; Olivera et al., 2015). In most of the prior taxonomic literature, the species *Conus genuanus* from the eastern Atlantic has been included in the *Kalloconus* clade (Cunha et al., 2005). However, as noted by Abalde et al. (2019), this was caused by a misidentification, and *C*. *genuanus* is more closely related to *Conus imperialis*. Despite this, both Abalde et al. (2019) and Tenorio et al. (2020) give *C*. *genuanus* the genus name *Genuanoconus*. We recently resolved this problem by using many further *Stephanoconus* sequences than are present in those trees, clearly embedding *C*. *genuanus* within the *Stephanoconus* (Figure 2) (Torres et al., 2021), an assignment that will be supported by additional biochemical data presented in this study. Although observations of prey capture are not available for every species within *Stephanoconus*, all of the records in the literature suggest that this group has specialized on amphinomid polychaetes as their major prey (a group widely known as fireworms, which have painful stinging bristles). This literature has been summarized by Kohn (Kohn, 2014).

The biodiversity of the genus *Conus* is generally richest in the Indo-Pacific marine province, which is not surprising, since this encompasses a vast tract of the tropical marine environment from the Hawaiian Islands to Madagascar. Notably, as Kohn has pointed out, *Stephanoconus* is an apparent exception to this generalization (Kohn, 2014). There were traditionally only two species of *Stephanoconus* recognized from the Indo-Pacific, *C*. *imperialis* and *Conus zonatus* and three Panamic species: *Conus archon*, *Conus bartschi*, and *Conus brunneus*. In contrast, the speciation in the Western Atlantic and the Caribbean province has been remarkable, and although there are differences of opinion regarding which forms are truly different species (compare the treatment of Kohn (2014) to Monnier et al. (2018), it seems that almost every island group has evolved its own endemic *Stephanoconus* species or subspecies (Kohn, 2014; Monnier et al., 2018). At present, *C*. *genuanus* is the only member of this clade known from the Eastern Atlantic/Mediterranean province.

All of the non-Indo-Pacific forms of *Stephanoconus* are found in relatively shallow-water marine environments, and have been collected by divers. There is however, one deep-water species group whose biology is poorly known, and these are represented by a set of relatively small Western Pacific species. The best characterized of these is *Conus chiangi* (Watkins et al., 2010). Other small deep-water species such as *Conus polongimarumai* and *Conus suduirauti* likely belong to this group as well. A curious feature of the phylogeny shown in Figure 2 is that *C*. *chiangi*, a deep-water Indo-Pacific species, does not appear to branch with the shallow Indo-Pacific species, suggesting that the divergence between deep and shallow-water Indo-Pacific *Stephanoconus* may have occurred before the ancestor of all shallow-water forms radiated out of the Indo-Pacific. Unfortunately, because of their relative rarity, and the difficulty in collecting these deep-water forms, little is known at the present time about their biology or biochemistry.

The division between deep and shallow-water groups can also be observed in the Indo-Pacific *Stephanoconus*. Two different populations comprise *C*. *imperialis*, which are genetically distinct enough that they may be different species (Torres et al., 2021). One of these occupies shallow, warmer waters, while the other appears to be a deeper-water specialist that may have radiated to cooler Indo-Pacific marine habitats.

## Non-Peptidic Small Molecules Reported from Cone Snail Venoms

**FIGURE 3:**
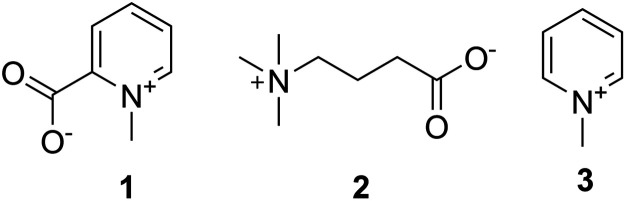
First small molecules identified in cone snails. These compounds were found by Kohn in 1960 in *C*. *textile*, *C*. *striatus*; **1** was identified in *C*. *litteratus*, *C*. *marmoreus*, and *C*. *magus*.

The first identification of non-peptidic venom components in cone snails was the work of Kohn et al. (1960) (Figure 3). The presence of homarine (**1**), γ-butyrobetaine (**2**), and *N*-methylpyridinium (**3**) in *Conus textile* and *Conus striatus* venom was established. *Conus marmoreus*, *Conus litteratus*, and *Conus magus* contained **1** as well. In this early work, it was proposed that small molecules might be important for prey capture, a prescient idea that was largely forgotten with the discovery of the conopeptides as the major active components in venom glands.

**FIGURE 4:**
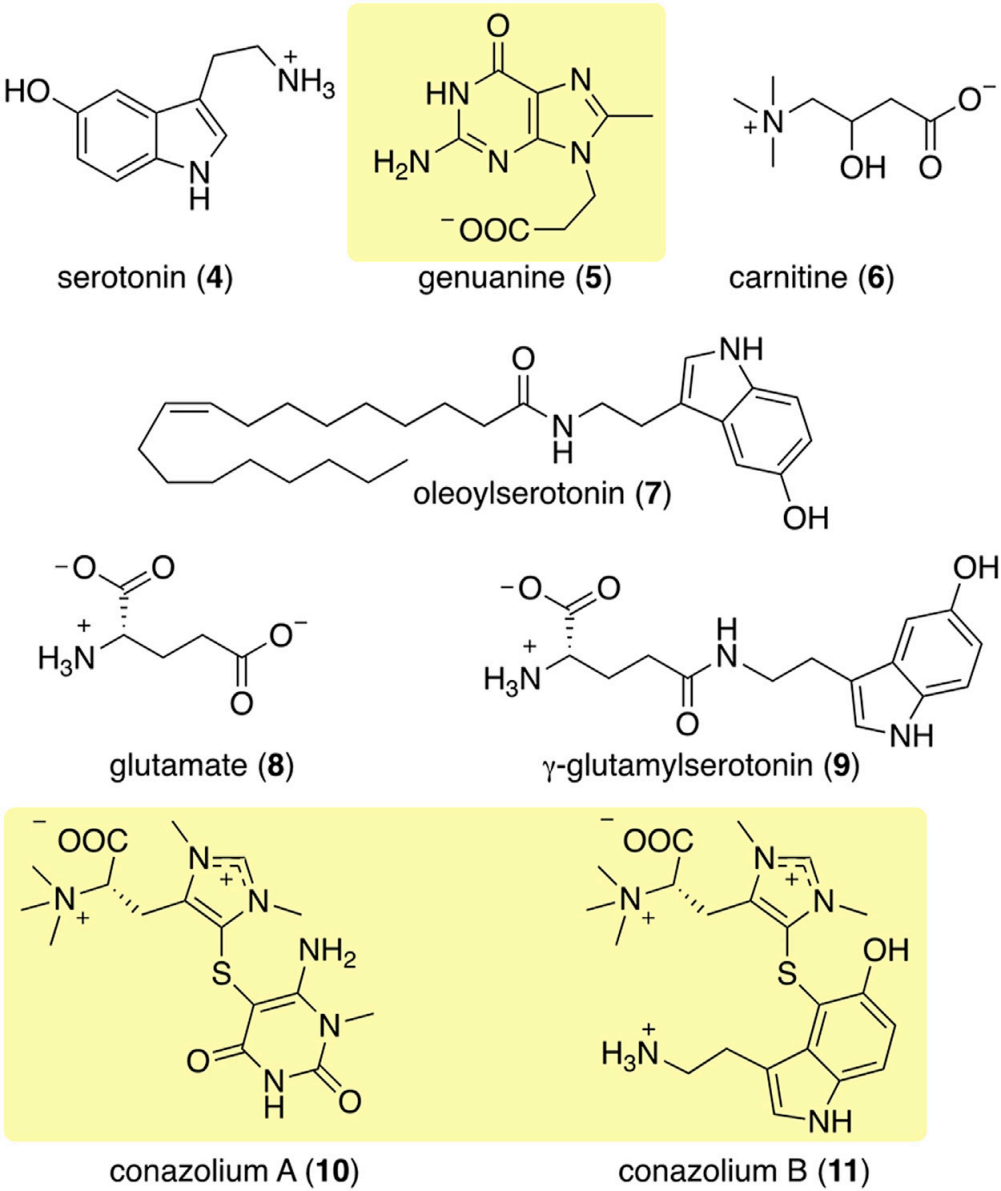
Some of the most abundant compounds discovered in the colored venoms of *Stephanoconus* snails. Those in shaded yellow are compounds so far found only in *Stephanoconus*.

Following Kohn’s pioneering work, a number of small molecule natural products have been observed in diverse cone snail venom glands and other tissues. The identification of serotonin (**4**) in the venom of *C*. *imperialis* (McIntosh et al., 1993) was the first report where a non-peptide venom component was purified using a bioactivity-based assay (Figure 4). However, the first novel non-peptidic compound with a new chemical structure was characterized relatively recently from *C*. *genuanus* (Neves et al., 2015). Genuanine (**5**), a derivative of guanine with some unique chemical features, had neuroactivity using several different assays.

Neves investigated the small molecules from *Conus genuanus* because he noticed that its venom gland had two different colors, each with different constituents (Neves et al., 2015). The proximal venom gland was the characteristic dull yellow found in most cone snails, and it was dominated by conopeptides. By contrast to most cones, but as is common in *Stephanoconus*, the distal venom gland was deep red in color. Instead of conopeptides, the gland contained mostly small molecules. Neves speculated that these might be important to predation because he observed that when *Conus genuanus* injects its venom into prey polychaetes, the red coloration from the distal venom gland can be readily followed from gland to prey body. The molecule responsible for red coloration has so far eluded structure determination.

Similarly, in a study of *Stephanoconus* feeding behavior, Kohn observed the red coloring from shallow-water *C*. *imperialis* as it was injected into prey polychaetes (Kohn and Hunter, 2001). Like *C*. *genuanus*, *C*. *imperialis* has a bifurcated venom gland, where the proximal end is yellowish while the distal end is deeply pigmented. Because of these similarities, *C*. *imperialis* chemistry was investigated and compared to that of *C*. *genuanus* in a metabolomics study (Torres et al., 2021). It should be noted that, at the time we did this study, the close phylogenetic relationship between *C*. *genuanus* and *C*. *imperialis* was not known. Like *C*. *genuanus*, the distal venom gland of *C*. *imperialis* contains almost solely small molecules, while the proximal venom gland contains mostly conopeptides. Shallow-water *C*. *imperialis* generally has a dark red distal venom gland, while its deep-water relative has a deep greenish distal gland. In keeping with this color difference, *C*. *imperialis* had notably different constituents depending upon whether the specimen belonged to the deep- or shallow-water clade, and *C*. *genuanus* harbored yet another set of compounds.

**FIGURE 5:**
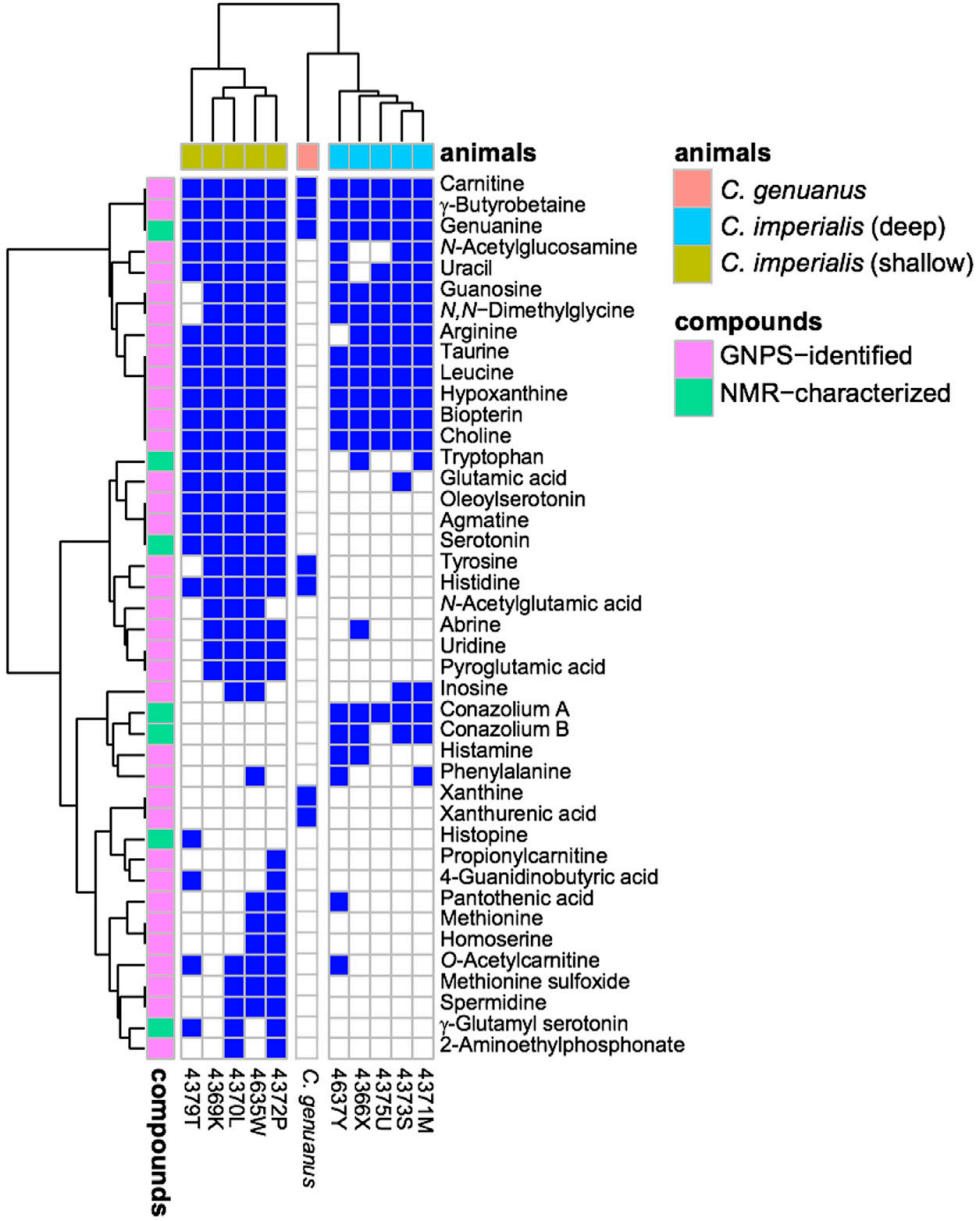
Small molecules in *Stephanoconus* are highly species specific. This chart indicates where each small molecule is found (blue), showing that the deep/shallow *C*. *imperialis* species and *C. genuanus* contain distinctly different small molecule chemistry in their colored venom glands. GNPS: Global Natural Product Social Molecular Networking.

Using mass spectrometry and nuclear magnetic resonance analyses, the only compounds held in common by all three types of cones were γ-butyrobetaine (**2**), carnitine (**6**), and genuanine (**5**) (Figures 4, 5). The small-molecule constituents of the three types of *Stephanoconus* were quite distinct. As previously shown (McIntosh et al., 1993), shallow-water *C*. *imperialis* contained the neurotransmitter serotonin (**4**), which was absent from deep-water specimens. The shallow-water specimens also tended to be rich in other neurotransmitters and their derivatives, such as oleoylserotonin (**7**), glutamate (**8**), and γ-glutamylserotonin (**9**) (Torres et al., 2021). These compounds were present in sufficient abundance that they would certainly exhibit activity in prey animals. Essentially, these are cocktails of diverse, neuroactive small molecules, several of which are potently active in annelids.

While they did not contain abundant serotonin or glutamate, the deep-water *C*. *imperialis* specimens had their own distinctive chemistry. In particular, they were dominated by abundant novel compounds, conazoliums A (**10**) and B (**11**) (Torres et al., 2021). The conazoliums are elaborate derivatives of the antioxidant, ovothiol, which is a common component in marine animals. Indeed, ovothiol and uracil are used by polychaetes as mating pheromones (Zeeck et al., 1996; Zeeck et al., 1998a; Zeeck et al., 1998b; Watson et al., 2000). Based upon that observation, Torres proposed that conazolium A (**10**) and the uric acid analog genuanine (**5**) might be used in a polychaete prey-capture strategy (Torres et al., 2021). The compounds are highly abundant, with for example **10** comprising ∼20% of the dry weight of the venom gland. Addition of conazolium A (**10**) to seawater containing female worms caused the worms to initiate mating behavior. When genuanine (**5**) was added to the seawater near male worms, it led to rapid sperm release. While the normal polychaete hormones are readily degraded both enzymatically and oxidatively, conazolium A (**10**) and genuanine (**5**) are likely stable to common metabolic routes. The resulting worm behaviors suggest a role for these small molecules in attracting prey, in luring them from their burrows, or in interfering with an unknown neuronal target.

In contrast to certain *Stephanoconus* species, other cones generally have venom glands with a single color, which are dominated by conopeptides. While performing bioassay-guided small molecule discovery, we found that in 20 species of cone snails the major compounds present were guanosine (**12**), and the monophosphates of guanosine (**13**) and adenosine (**14**), and often homarine (**1**). These types of compounds are found in *C*. *imperialis* (Torres et al., 2021) and *C*. *genuanus* (Neves et al., 2015) as part of a more complex cocktail, but in other species they are the most abundant small molecules (Table 1, unpublished data). Since purinosines are signaling molecules involved in pain and other sensory transmission (Burnstock, 2007), it is possible that such components could be involved in predation, although this has yet to be investigated. Purines are involved in spider venoms, for example (Schroeder et al., 2008). An early interpretation of these data, awaiting further work, is that outside of *Stephanoconus*, small molecules may not be important contributors to venoms.

**TABLE 1:** The most abundant purine derivatives observed in *Conus* venom glands (plus = present; minus = absent)^a^.

Subgenus	Species	Compound Number			
		5	12	13	14
*Stephanoconus*	*C*. *imperialis* (shallow)	+	+	+	+
	*C*. *imperialis* (deep)	+	+	+	+
	*C*. *genuanus*	+	+	+	+
*Dendroconus*	*C*. *betulinus*	−	+	+	+
*Puncticulus*	*C*. *caracteristicus*	−	+	+	+
*Gastridium*	*C*. *geographus*	−	+	+	+
*Conus*	*C*. *marmoreus*	−	+	+	+
*Pionoconus*	*C*. *striatus*	−	+	+	+
*Asprella*	*C*. *sulcatus*	−	+	+	+
*Virgiconus*	*C*. *virgo*	−	+	+	+

^a^ For *Stephanoconus* species listed here, genuanine (**5**) is the major component. Other, more common purines (**12**–**14**) from primary metabolism are relatively minor in *Stephanoconus*, but represent the major purines in other cone species.

**FIGURE 6:**
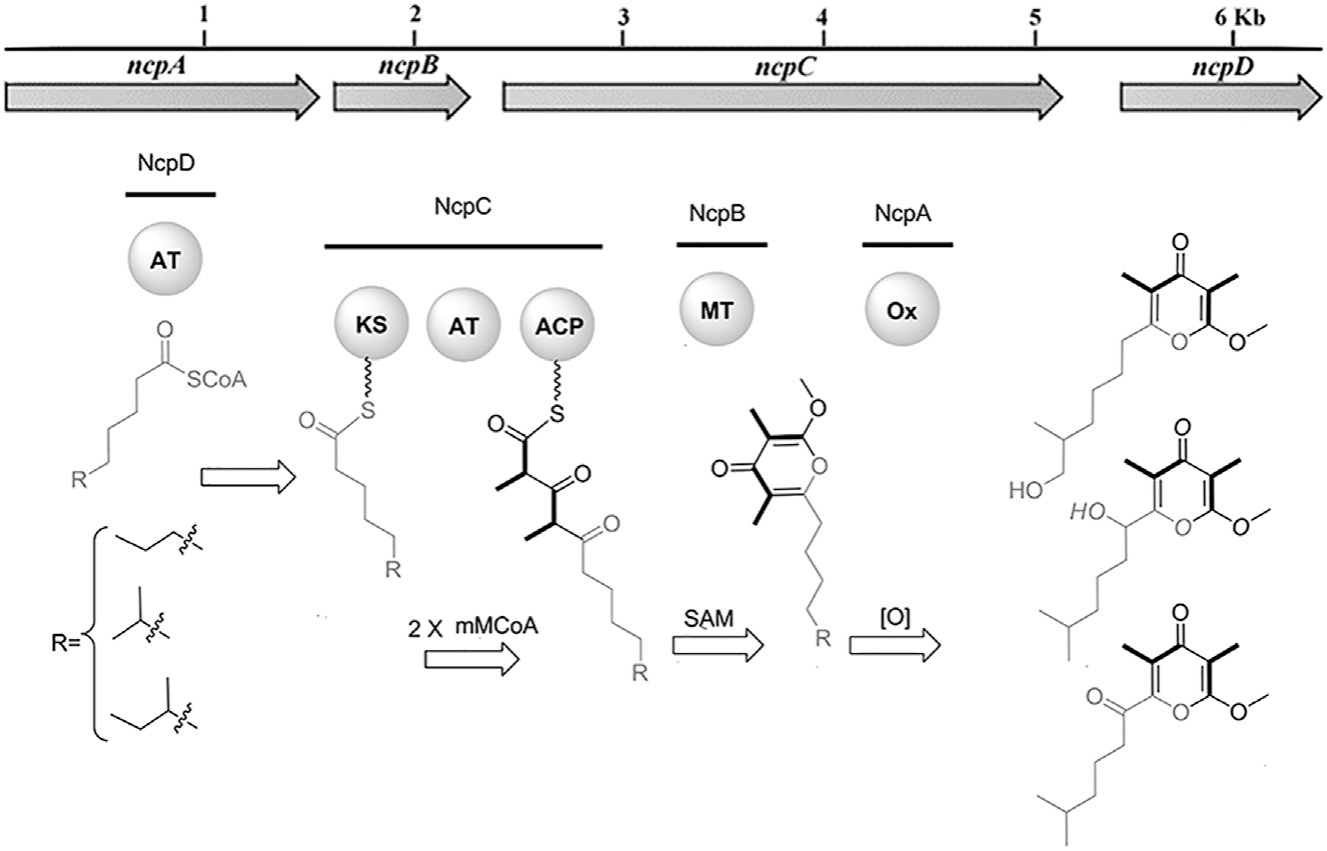
Bacterial biosynthesis of nocapyrones. A genetic locus in *Nocardiopsis* sp. bacteria is responsible for producing ion channel modulators found in the whole snails, *C. rolani* and *C. tribblei*. Bold wedges within chemical structures indicate incorporation of methylmalonate. Figure from Lin et al. (2013).

In recent years, the role of the microbiome in shaping animal chemistry has been increasingly appreciated (Morita and Schmidt, 2018). Indeed, marine animals offered some of the first examples where symbiotic bacteria synthesize molecules isolated from the whole animals. In one study of cone snail symbionts, bacteria were cultivated from *Conus rolani* and *Conus tribblei* (Lin et al., 2013). Novel neuroactive pyrones, the nocapyrones, were isolated from the bacteria (Figure 6). Subsequently, the same neuroactive compounds were found concentrated in the mucus secretions and in the venom glands of the cones. These were present in sufficient concentrations to modulate neurons in prey animals. The available data suggested that they likely modulate the activity of sodium channels, although the precise targets are not known.

Small molecules have also been identified in other cone snail tissues beyond the venom gland. Several unusual cholesterol derivatives were found in whole body extracts (which included the venom glands) from *Conus pulicarius*, *Conus leopardus*, *Conus ebraeus*, and *Conus tessulatus* (Aknin et al., 1998; Lee et al., 2017).

In summary, although there is still a paucity of literature in the area, current data show that at least some cone snail venom glands are rich in small-molecules, including novel natural products. Many of these are neuroactive in various models, and some directly target the hormonal systems of prey polychaetes. A majority of compounds isolated so far are alkaloids, betaines, neurotransmitters, or purines, with only a small number of non-nitrogenous compounds described. Most of the interesting novel compounds so far found have been concentrated in the single subgenus, *Stephanoconus*, suggesting that members of this group will be a rich source for further discovery.

## Biosynthesis of Cone Snail Venom Small Molecules

The conopeptides that are the primary constituents of most cone snail venoms are synthesized in epithelial cells lining the venom gland (Buczek et al., 2005; Safavi-Hemami et al., 2011). Of the biosynthetic mechanisms that are known, they fall squarely into standard eukaryotic metabolic space, and they are encoded in the snail genomes (Arnison et al., 2013). Similarly, of the components so far discovered in cone snail venoms, most are products of common eukaryotic metabolism, and thus are likely made in the venom glands. A few, such as γ-glutamylserotonin (**9**), are relatively restricted in phylogenetic distribution. γ-Glutamylserotonin **(9**) is found in annelids and in the gastropod mollusc *Aplysia californica*, where it was shown to be an enzymatic product of serotonin (**4**) catabolism (McCaman et al., 1985; Sloley, 1994; Stuart et al., 2003). A few of the *Stephanoconus* products are specialized metabolites found so far only in those cones: genuanine (**5**) and the conazolium (**10**–**11**) family of alkaloids. Genuanine (**5**) consists of guanine that has been *C*-methylated, with the addition of *N*-propionate. The latter is an unprecedented modification in nature, but both the *N*- and *C*-modifications are reminiscent of radical-mediated chemistry (Grove et al., 2011).

**FIGURE 7:**
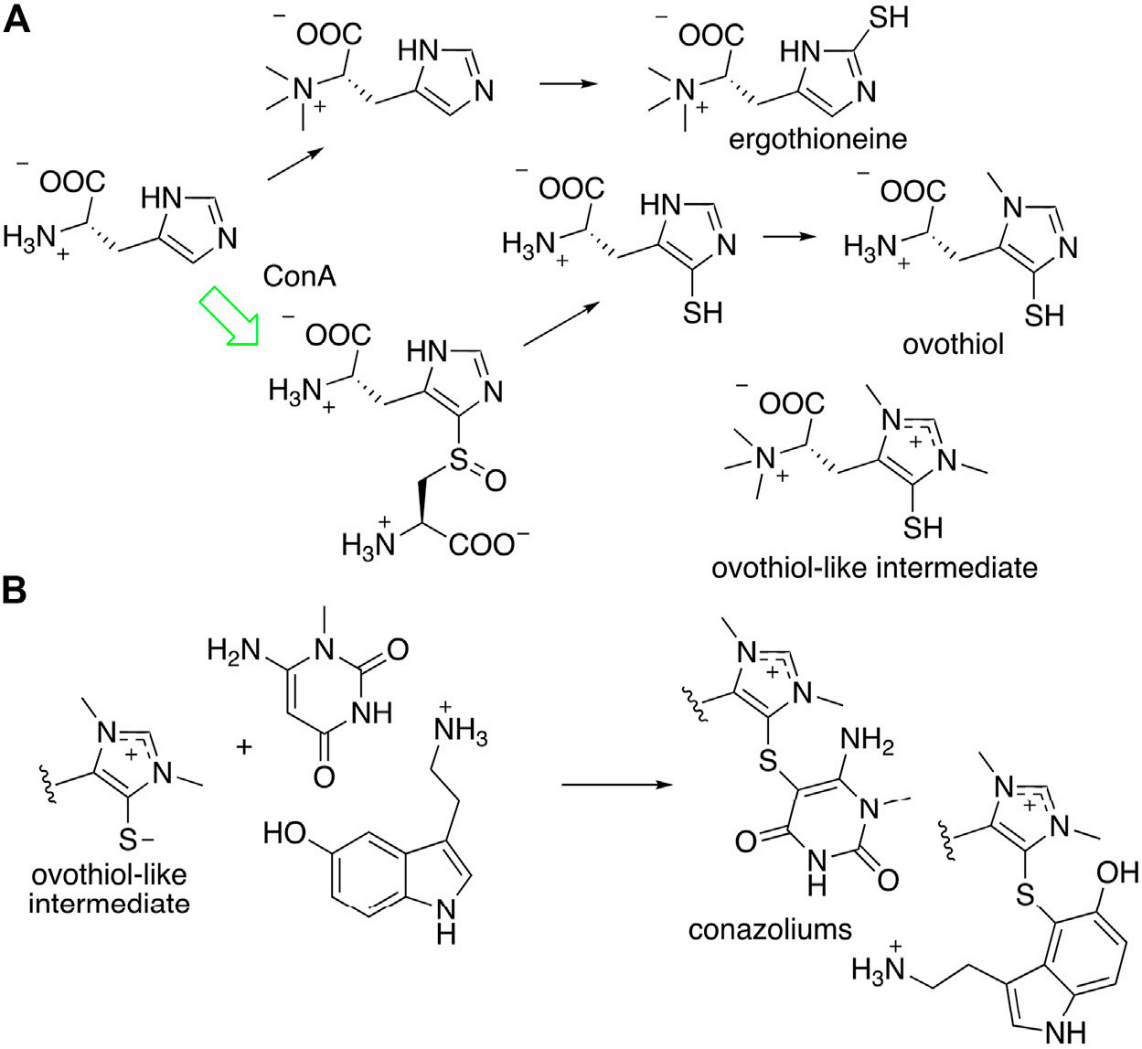
Proposed biosynthesis of conazolium. **(A)** Known routes to antioxidants ergothioneine and ovothiol. The synthesis of an “ovothiol-like intermediate” that is the precursor to conazoliums has features of both pathways, but with an additional methylation on the imidazole ring. The arrow in green indicates a reaction that was characterized using the *C. imperialis* enzyme, ConA. **(B)** The “ovothiol-like intermediate” reacts with electrophilic metabolites to create the conazoliums (**10**–**11**).

Conazoliums (**10**–**11**) are derived from a combination of an ovothiol-like alkaloid and an aromatic residue (Figure 7) (Torres et al., 2021). The ovothiol-like side has an unprecedented combination of features. The alpha amine is trimethylated, which is as found in ergothioneine, whereas the imidazole head group is thiolated in the ovothiol position. Both imidazole nitrogens are methylated, forming an imidazolium cation that may have some carbene character. This feature is found in several natural products from marine animals. The biosynthesis of ovothiol has been studied; in animals, the first reactions in the biosynthetic pathway are genetically conserved, but later reactions are not, and for the most part remain unknown. An *ovoA*-like gene, *conA*, is expressed in the colored venom gland of *C*. *imperialis*. The ConA protein was produced recombinantly in *Escherichia coli* bacteria and purified to homogeneity. When it was treated with the normal substrates of OvoA, ConA produced the expected product, a known intermediate on the ovothiol pathway. Thus, it was shown that the venom glands of *C*. *imperialis* have the capacity to synthesize the precursor of conazoliums (Torres et al., 2021).

Ovothiol and the related antioxidant ergothioneine have highly nucleophilic sulfur residues that detoxify cells during oxidative stress by removing reactive electrophilic species (Castellano and Seebeck, 2018). In the case of conazoliums (**10**–**11**), the reaction between the ovothiol-like moiety and the aromatic substituents are at the most electrophilic carbon in the molecule. Thus, it is possible that this reaction may even be non-enzymatic. In conazolium A (**10**), an ovothiol-like moiety has reacted with aminomethyluracil, a compound not previously found anywhere in nature, while in conazolium B the uracil moiety has been replaced with serotonin. Natural products featuring ovothiol or its close congeners conjugated with an electrophilic second species have been identified in octopus, sponges, and echinoderms (Castellano and Seebeck, 2018).

Overall, most of the specialized natural products found in cone snail venoms are likely to be made there, using the eukaryotic enzymes in cells lining the venom gland. An exception to this trend may be found in the nocapyrone polyketides, found in both the mucus and the venom glands of *C*. *rolani* and *C*. *tribblei*. The biosynthetic locus was identified not in the snails, but in bacteria living within the snails (Figure 6; Lin et al., 2013). To provide evidence that the correct locus was identified, the bacterial methyltransferase was expressed in *E*. *coli*, and the purified enzyme was used in a reaction with the native pyrone substrate, performing the predicted reaction. An unusual polyketide synthase was present in the bacterial locus, but it has not been further investigated.

The specialized metabolites found in cone snail venoms, whether eukaryotic or bacterial in origin, represent a resource for discovering new compounds and biochemical modifications. In the case of conazoliums, for example, although the core structure is derived from the common metabolite ovothiol, the additional decorations derive from novel biochemical adaptations. It will be of great interest to determine the origin and evolution of these novel compounds and biochemical reactions and the molecular adaptations that take place during their recruitment into the venom gland. In the evolution of venom insulins in cone snails, duplication of an ancestral insulin gene generated two copies, one that retained its endogenous signaling function and another one that experienced neofunctionalization and diversification upon its recruitment into the venom gland (Safavi-Hemami et al., 2016b). Similar mechanisms were shown for enzymes that are important for conopeptide biosynthesis. For example, duplication of an ancestral protein disulfide isomerase (PDI) gene gave rise to a large family of conopeptide-specialized PDIs (csPDIs) that rapidly evolved and diversified to assist in the folding of diverse conopeptide structural disulfide scaffolds (Safavi-Hemami et al., 2016a). It is possible that a similar mechanism comes into play in the origin of venom small molecules (specifically, in the origin of their biosynthetic enzymes), although this has yet to be investigated.

## Pharmacology of Cone Snail Venom Small Molecules

The peptide components of cone snail venoms are well known, and their pharmacological properties have been studied for decades (Olivera et al., 2014). By contrast, our understanding of cone snail small molecule pharmacology is in its infancy. Many of the previously described compounds found in cone snail venoms are either neurotransmitters, close relatives of neurotransmitters, or their activities on neurons have been well characterized. Nocapyrones, found in some cone snails in various tissues including venom ducts, are potent modulators of mouse dorsal root ganglion neurons, with IC_50_s around 2 μM (Lin et al., 2013).

Only two compounds found so far are unique to cone snail venom ducts and are present in sufficient quantities to perform pharmacological studies; these compounds (genuanine (**5**) and conazolium A (**10**)) both have neuromodulatory effects. At a dose of 40 nmol/mouse, genuanine (**5**) paralyzed mice when injected intracranially (Neves et al., 2015). Paralysis was fully reversible after a period of about 2 h. The molecular target leading to paralysis is so far not known. By contrast, conazolium A (**10**) had no obvious effects upon intracranial injection or when loaded onto primary cultures of DRG neurons. Instead, **10** inhibited the human α7 nicotinic acetylcholine receptor with an IC_50_ of 24.4 μM (Torres et al., 2021). This assay was attempted because of the distant resemblance of **10** to urochanycholine (see below), making it likely that this is not the most pharmacologically relevant target. Instead, these findings provide proof-of-concept that, as found with the many well-characterized cone snail venom peptides, the small molecules also exhibit activity on neurons or neuronal targets. These results suggest that cone snail venom small molecules may provide rich sources for further discovery.

## Context and Future Directions

The presence of small molecules in cone snail venoms is far from unique, but is rather a property of diverse venomous animals. Animals such as insects and spiders often contain alkaloids, neurotransmitters, and nucleoside derivatives as major venom components. Although the constituents differ from what we have found in cone snails, the overall pattern is quite similar. What is not always completely clear from the literature is the biological roles that the compounds perform in nature.

Of more interest to drug discovery are the unique, specialized metabolites that are found only in a few species or groups of organisms. These compounds are often highly evolved for specific targets or ecological niches. Thus far, most work has been done in ants and spiders, where unique compounds with human disease-treating potential have been discovered. For example, fire ant-derived solenopsins have been investigated for their potential in treating psoriasis, while spider polyamines have been examined for treating pain and other conditions (Estrada et al., 2007; Arbiser et al., 2017). These and many other alkaloids from spiders and insects are potent, toxic components of offensive/defensive systems (Touchard et al., 2016). Additionally, hydroid and jellyfish venoms are rich in unique small molecules, at least some of which are involved in chemical defense (Stachowicz and Lindquist, 2000; Lindquist, 2002; Reinicke et al., 2020).

Gastropod molluscs have been extensively studied for their bioactive and defensive small molecules, although with little focus on venoms. Much of the work has focused on soft-bodied molluscs. For example, dorid nudibranchs contain diverse small molecules that defend the shell-less molluscs from predation (Faulkner, 1992). Some molluscs, such as *Navanax*, engage in chemical communication and signaling using small molecules (Sleeper and Fenical, 1977). In addition, many molluscs famously bioaccumulate various types of toxins, presumably for defense.

Within the shelled gastropods, data on non-venom compounds have been recently reviewed (Turner et al., 2018). What is evident is that many tissues beyond the venom gland are critical in the chemical ecology of shelled gastropods. For example, a number of choline derivatives, especially urocanycholine (murexine), are secreted from the salivary glands of muricid molluscs (Erspamer and Glasser, 1957; Roseghini et al., 1996). Urocanycholine was briefly under study as a potential therapeutic muscle relaxant because of its nicotinic acetylcholine receptor blocking properties. The hypobranchial gland has also emerged as the locus of defensive compounds. For example, a brominated tryptamine betaine, isolated from the hypobranchial gland defensive secretion of *Calliostoma canaliculatum*, inhibits potassium channels (Kelley et al., 2003). Both murexine and tyrian purple are likely synthesized in the hypobranchial glands of muricids, and subsequently distributed to target tissues where they play different biological roles (Rudd et al., 2015).

However, in contrast to the arthropods where venom small molecules have been intensively studied, relatively little is yet known about the small molecule contribution to gastropod venoms. A few exceptions exist: for example, the blue-ringed octopus envenomates prey with tetrodotoxin (Sheumack et al., 1978). Here, we describe how cone snails, well known for their diverse peptide toxins, also contain small molecules that are used in hunting, and possibly in defense. In particular, the colored venoms of *Stephanoconus* have proved to be a rich source, although even from *Stephanoconus* only a few species have been explored. Most of the >40 cone snail sub-genera comprising the majority of species have not yet been examined. Since there are likely to be hundreds of thousands of venomous animals, the rich known examples of venom small molecules represent just a small part of the chemical potential from venoms. Thus, further examination of these species should yield new bioactive compounds that are used in nature and that might have relevance to human medicine.

One of the major limitations of looking at venom chemistry has been the very small sample sizes available, necessitating cutting-edge instrumentation and a relatively large number of venom glands in order to characterize chemistry. The ability to obtain sufficient biomass has been greatly improved by technological advances in collection such as *lumum*-*lumun* and tangle nets, methods invented and deployed by Filipino fishermen to collect relatively rare specimens for the collectors trade (Seronay et al., 2010). Another advance is a return to the biology-first approach to drug discovery. In cones, complex hunting strategies are used, where both the behaviors and the chemicals used are highly elaborate and specialized to their habitats (Olivera et al., 2015; Olivera et al., 2017). Conazolium A and genuanine were discovered by chemistry first, but later their role in subduing prey was elucidated. By following the unique behaviors of different cone snail species, we believe that this process could be greatly accelerated.
